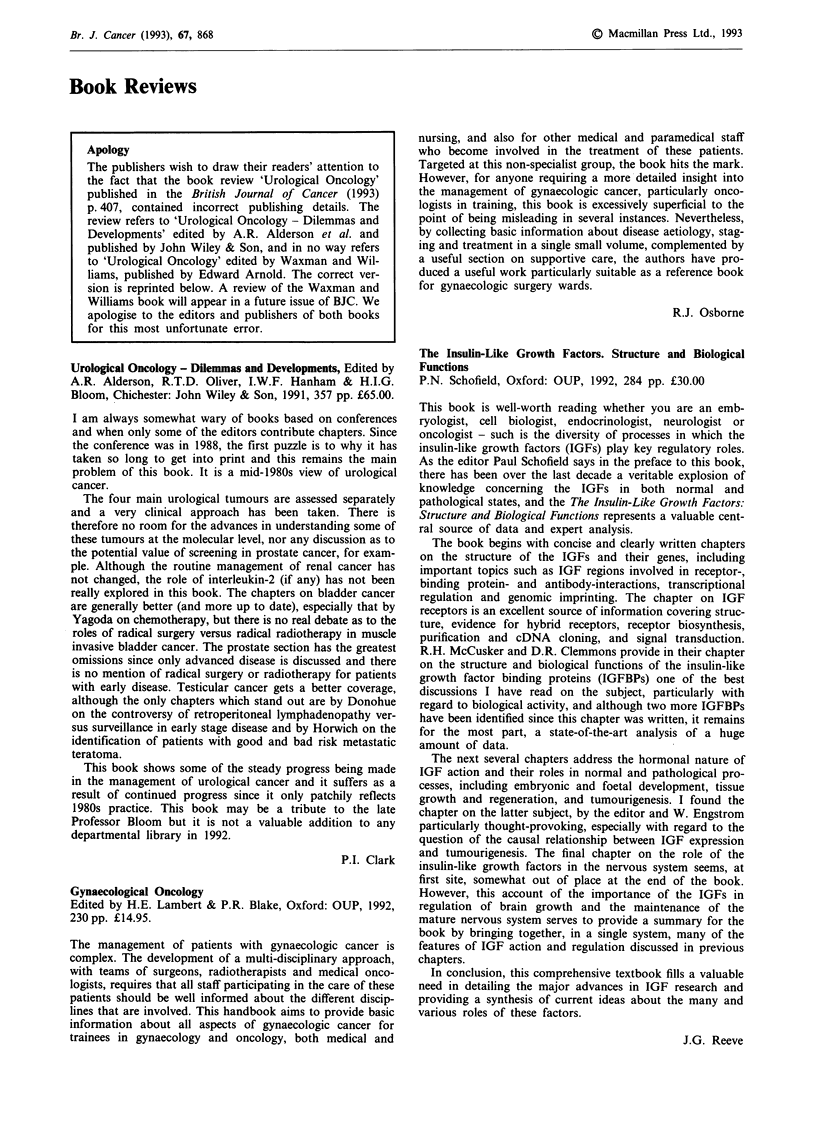# Gynaecological Oncology

**Published:** 1993-04

**Authors:** R.J. Osborne


**Gynaecological Oncology**
Edited by H.E. Lambert & P.R. Blake, Oxford: OUP, 1992, 230 pp. £14.95.

The management of patients with gynaecologic cancer is complex. The development of a multi-disciplinary approach, with teams of surgeons, radiotherapists and medical oncologists, requires that all staff participating in the care of these patients should be well informed about the different disciplines that are involved. This handbook aims to provide basic information about all aspects of gynaecologic cancer for trainees in gynaecology and oncology, both medical and nursing, and also for other medical and paramedical staff who become involved in the treatment of these patients. Targeted at this non-specialist group, the book hits the mark. However, for anyone requiring a more detailed insight into the management of gynaecologic cancer, particularly oncologists in training, this book is excessively superficial to the point of being misleading in several instances. Nevertheless, by collecting basic information about disease aetiology, staging and treatment in a single small volume, complemented by a useful section on supportive care, the authors have produced a useful work particularly suitable as a reference book for gynaecologic surgery wards.

R.J. Osborne